# Transformative medical education: must community-based traineeship experiences be part of the curriculum? A qualitative study

**DOI:** 10.1186/s12939-020-01213-4

**Published:** 2020-06-10

**Authors:** Julie Massé, Sophie Dupéré, Élisabeth Martin, Martine C. Lévesque

**Affiliations:** 1 VITAM, Centre de recherche en santé durable, Pavillon Landry-Poulin, 2525, chemin de la Canardière, Quebec city, Québec G1J 0A4 Canada; 2 Faculté des sciences infirmières de l’Université Laval, Pavillon Ferdinand-Vandry, 1050, avenue de la Médecine, Université Laval, Quebec city, Québec G1V 0A6 Canada; 3 Vice-décanat à la pédagogie et au développement professionnel continu, Faculté de médecine de l’Université Laval, Pavillon Ferdinand-Vandry, 1050, avenue de la Médecine, Université Laval, Quebec city, Québec G1V 0A6 Canada; 4 Centre de recherche de Montréal sur les inégalités sociales, les discriminations et les pratiques alternatives de citoyenneté, 66 rue Sainte-Catherine Est, Montréal, Québec H2X 1K7 Canada; 5 École de réadaptation de l’Université de Montréal, Faculté de médecine, Université de Montréal, 7077 avenue du Parc, Montréal, Québec H3N 1X7 Canada

**Keywords:** Transformative learning, Reflexive practice, Social vulnerability, Community-based, medical education, access-to-care equity, health equity

## Abstract

**Background:**

There are shortcomings in medical practitioners’ capacity to adapt to the particular needs of people experiencing circumstances of social vulnerability. Clinical traineeships create opportunities for the acquisition of knowledge, competencies, attitudes, and behaviors. However, some authors question the learnings to be made through classical clinical training pathways. This article explores the learnings gained from a traineeship experience within a community-based clinical setting intended for patients experiencing social vulnerability and operating under an alternative paradigm of care. To our knowledge, there is little research intended to identify and understand what medical trainees gain from their experience in such contexts.

**Methods:**

This exploratory qualitative study is based on twelve interviews with practicing physicians who completed a traineeship at *La Maison Bleue* (Montreal, Canada) and three interviews conducted with key informants involved in traineeship management. Based on Mezirow’s theory of transformational learning, data were analyzed according to L’Écuyer’s principles of qualitative content analysis. NVivo software was used.

**Results:**

The main learnings gained through the traineeship are related to (1) greater awareness of beliefs, assumptions and biases through prejudice deconstruction, cultural humility and critical reflection on own limitations, power and privileges; (2) the development of critical perspectives regarding the health care system; (3) a renewed vision of medical practice involving a less stigmatizing approach, advocacy, empowerment, interdisciplinarity and intersectorality; and (4) strengthened professional identity and future practice orientation including confirmation of interest for community-based practice, the identification of criteria for choosing a future practice setting, and commitment to becoming an actor of social change. Certain characteristics of the setting, the patients and the learner’s individual profile are shown to be factors that promote these learnings.

**Conclusions:**

This article highlights how a traineeship experience within a clinical setting intended for a clientele experiencing circumstances of social vulnerability and operating under an alternative paradigm presents an opportunity for transformative learning and health practice transformation toward renewed values of health equity and social justice. Our findings suggest medical traineeships in community-based clinical settings are a promising lead to foster the development of fundamental learnings that are conducive to acceptable and equitable care for people experiencing social vulnerability.

## Background

It is generally recognized that the incidence of many health-related issues such as chronic disease, injuries and infant mortality in developed countries follows a social gradient according to which the lower an individual’s socio-economic position is, the more likely he or she is to be in poor health [1, 2]. This implies that some individuals or sub-populations are exposed to difficult social, economic and environmental conditions that may ultimately result in health problems [3]. Health inequities refer to those differences in health that are unnecessary, avoidable, unfair and unjust [4]. While several studies have found that such observed health inequities are mainly due to determinants external to health systems, it must be recognized that barriers experienced in accessing care (and especially front-line care) can play a major role in increasing these inequities [5].

In Quebec,^1^ access-to-care barriers (on a geographical, social or physical level) are detrimental to populations living in situations of social vulnerability, such as low-income people, people with disabilities, members of Indigenous communities, recent immigrants and refugees in precarious migration situations [7, 8]. Despite efforts to reform the health care system to generally improve the correspondence between the population’s needs and the supply of care, one observation emerges: social inequalities in health remain poorly addressed in public policy, system design and health practices [9]. Consequently, the health gains resulting from the efforts made at all these levels seem to benefit individuals and social groups unequally [10] and unfairly, thus fostering the persistence of health inequities.

Authors suggest such inability to address inequities results from the tendency of reform initiatives to focus on tackling factors contributing to patients’ potential to access health care [11, 12] such as approachability, availability, accommodation or affordability [13]. However, it is increasingly recognized that acceptability and appropriateness of care [13] may have significant repercussions on actual recourse to care, especially for people experiencing circumstances of social vulnerability. Those repercussions are put forward in studies showing gaps in the capacity of medical practices to foster equitable access. For example, when people experiencing circumstances of social vulnerability use health care, the services offered often don’t meet their perceived needs [14]. Furthermore, studies reveal the effects of perceived negative experiences of care (e.g. feeling misunderstood, discredited, despised, judged, stigmatized) on future recourse-to-care decisions [15, 16].

This exposes the relevance of considering both the demand and the supply of care in addressing cultural and social determinants of access when designing and implementing front-line initiatives aimed at addressing social inequities in health. Following Levesque and colleagues [13], this new focus implies, on one hand, considering people’s capacity to seek care (e.g. issues of health literacy, personal autonomy, capacity and liberty to choose to seek care, knowledge about health care options and individual rights) and, on the other hand, a reflective analysis of health care aspects linked to acceptability. Accordingly, the emerging challenge involves, for health professionals, the need for (a) renewed knowledge and understanding of health issues resulting from social vulnerability and (b) the acquisition of competencies and abilities required to provide acceptable care to people experiencing circumstances of social vulnerability.

As they allow direct contact with real patients, clinical traineeships create opportunities for future physicians’ acquisition of particular knowledge, understandings, competencies, attitudes, and behaviors [17]. However, given the pervasive medical hierarchy and dominant biomedical paradigm imposing certain ways of understanding, questioning and solving health problems (e.g. following a mechanistic and deterministic rationale, fostering a pathogenic and realistic conception of health), some authors call into question the learning gained from classical clinical training [18–20]. Likewise, Taylor and Wendland [21] suggest that learners, through traditional clinical training, « gradually learn to recognize bodily pathologies in their patients and to *unsee* (as irrelevant) the social pathologies and institutional structures that are powerful predictors of health and disease » (p.55). It’s worth noting that some efforts have been made following the Commission on Social Determinants of Health’s recommendation to make social determinants of health a standard and compulsory part of training of medical and health professionals [2]. Notably, the Royal College of Physicians and Surgeons of Canada’s educational framework (CanMEDS) describes, among the abilities required of physicians to effectively meet health care needs, the Health Advocate role. This role recognizes that improving health implies, for physicians, a responsibility to promote health equity and to respond to the needs of vulnerable or marginalized populations [22]. However, given that the entire medical curriculum’s primary focus remains fundamentally biomedical, curative and individualist, an ongoing and important gap is observed in the way the Health Advocate role is concretely taught and learned through medical training [23]. Moreover, research on this specific topic is scarce.

A way forward for renewed clinical training opportunities lies in the promise of community-based clinical settings operating under an alternative paradigm—that which embraces emic, constructivist and interpretivist perspectives open to the singularity of the patient’s experience and life context. Such alternative settings promote and foster a more positive and holistic conception of health. Against the major challenges of front-line care as a backdrop, such alternative organizational experiences have gradually emerged in Quebec. They aim to reduce the access-to-care inequalities impacting certain social groups experiencing circumstances of social vulnerability. These organizations are often operating under a non-profit status, sometimes combined with mechanisms linking them to the formal health and social services system [24–26]. They generally present with a distinct culture, values, and purposes that foster collaborative practice and a certain flexibility in professional roles [27], together with a diversity-sensitive approach to care [28]. As these organizations often welcome medical students throughout their training, one might ask what are the repercussions of traineeships in those settings on the medical learner’s journey?

This article presents the results of a qualitative study exploring physicians’ perceptions of learnings gained through traineeship experience within a community-based clinical setting intended for patients experiencing social vulnerability and operating under an alternative paradigm of care in Montreal, Quebec, Canada. It also documents the characteristics of the traineeship experience that influenced their learnings. Moreover, the article discusses the potential health practices transformations associated with the learning opportunities offered by such settings operating under an alternative paradigm and their role for improving access-to-care and health equity. Thus, as a complement to the quantitative corpus available in the literature, this article presents added value for research and practice as it proposes an in-depth understanding of the “how’s” and “why’s” of the learnings observed.

### Medical trainee teaching and learning in non-traditional settings

Though we know little about the learning effects of community-based settings, the literature identifies several ways in which other types of non-traditional clinical training environments might positively impact medical trainees. Within these studies, we identified three distinct types of impact. Firstly, relations between the learning experience and a renewed vision of oneself are suggested. For instance, self-confidence and self-esteem may be positively impacted due to the feeling of making a significant contribution to communities’ well-being [29, 30]. A potential impact on the development of professional identity is also outlined, as professional objectives and career orientation may be influenced [31, 32]. Secondly, studies highlight the contribution to a renewed vision of the medical practice and profession. Notably, the reiteration of idealism and enthusiasm toward the medical profession is mentioned [33] in addition to a shift in the vision of front-line care and the expected characteristics of the patient-doctor relation [30, 34]. Thirdly, authors emphasize the impact of the learning experience on trainees’ perception of others and their reality. For instance, some suggest a renewed understanding of health determinants and their repercussions on people’s life courses and health trajectories [34]. Also, an impact on the valuation of collaborative work, communication, and leadership is suggested [35–38]. Moreover, repercussions on clinical practice are highlighted through the adoption of a congruent, competent, human and holistic approach to care [30, 39, 40] and an evolution in the way learners perceive, communicate and interact with patients [34]. An improved ability to adequately refer patients to needed resources is also outlined [33]. That being said, most studies report on the repercussions of non-traditional traineeship experiences in rural settings or in developing countries. Also, few studies privilege a qualitative methodology, thus limiting our in-depth understanding of the learning processes at work. Furthermore, the few studies cited mainly considered experiences in US clinical settings, limiting their applicability to other cultural, social, political and economic contexts.

## Methods

### Research setting

Our research setting is *La Maison Bleue*, a community-based front-line organization offering perinatal care to women and families experiencing circumstances of social vulnerability in Montreal, Quebec, Canada.^2^ Based on values of respect and openness, *La Maison Bleue*’s bio-psycho-social approach to care fosters empowering and supportive professional practices that mobilize patients’ personal and collective resources and adapt to their cultural realities and familial trajectories [25]. Accordingly, the definition of ‘vulnerability’ adopted by *La Maison Bleue* remains flexible and loosely defined, allowing for professional judgment in assessing needs on a “case-by-case” basis [25]. The potential vulnerability factors taken into account are numerous and may include: a precarious financial situation, low education, unwanted or teenage pregnancy, social isolation, mental health or addiction problems, abuse, neglect or violence, precarious migratory status or a difficult migration path, the involvement of the Youth Protection Department, single parenthood or marital difficulties. Interdisciplinarity is considered the linchpin of *La Maison Bleue*’s approach to care. Thus, a non-hierarchical partnership model of shared responsibility for care (in which the doctor is not considered as the central actor) is privileged [25]. Care is thus provided by an interdisciplinary team including physicians, nurses, social workers, midwives, psycho-educators and special educators. Furthermore, in line with our research objectives, it’s worth noting that training and teaching are central to *La Maison Bleue*’s organizational mission. Accordingly, several medical students, residents and medical fellows experience its approach through traineeships every year.

### Theoretical perspective

Mezirow’s Theory of transformational learning [41–43] guided our global understanding of adult learning as a multiphasic process triggered by a perceived dissonance between an experienced situation and usual frameworks of interpretation (a disorienting dilemma) and by which the learner modifies old thought patterns and perspectives through new experiences.

### Epistemological perspective and research design

The study reported rests on a constructivist epistemological paradigm [44, 45]. Given our interest in perceptions, experiences, and practices of actors in context, a qualitative methodology was privileged [46]. We opted for an exploratory and descriptive design as the research aims to clarify an ill-defined problem by producing knowledge on an insufficiently known phenomenon [47].

### Participants selection and recruitment strategies

To reach a participation rate allowing theoretical saturation to be achieved, the inclusion criteria were not very restrictive. Thus, participants are licensed front-line physicians (*n* = 10) and residents (*n* = 2) who completed a traineeship at *La Maison Bleue* during their medical training and were actively practicing within a Canadian primary care organization at the time of the research. Recruitment involved an email request submitted to *La Maison Bleue*’s former trainees (from lists maintained by *La Maison Bleue* and some of its partners) and complementary snowball techniques by which participants were asked to recommend physicians responding to inclusion criteria. An electronic form was developed to allow the identification of interested physicians consenting to be contacted for the next steps of their potential participation. It is therefore through this form that interested physicians provided their contact information.

Three key informants involved in traineeship management and logistics (from *La Maison Bleue*, the Quebec health and social services network and the academic community) were also recruited through purposeful sampling.

### Data collection strategies

The previously mentioned electronic form was also used to collect information that helped to profile potential participants. In addition to contextualizing the interviews, the information was intended to be used for a more focused analysis strategy targeting variations in participants’ discourse. Otherwise, data collection mainly relied on 12 semi-structured interviews with recruited physicians and residents. An interview guide was developed, in line with research objectives, to collect participants’ narratives of their traineeship experience at *La Maison Bleue*, and to identify learnings gained and characteristics of the experience that influenced these learnings. Consequently, we planned a set of questions with fairly broad formulations to allow participants to tell their stories and speak out about what was particularly important to them (e.g., Could you tell me about your traineeship experience at *La Maison Bleue*?; What did you gain concretely from this traineeship experience?; To what do you attribute this learning?). An interview guide was also specifically adapted for key informants. It was structured to collect information about (a) characteristics of the traineeships offered at *La Maison Bleue* (e.g.: How is the allocation of traineeships to medical students articulated administratively and logistically between CIUSSSs, Faculties of Medicine and *La Maison Bleue*?; What are the different categories of medical traineeships offered at *La Maison Bleue,* and how is each one set up in terms of duration and supervisory arrangements) and (b) respondents’ perception of the main learnings gained from a medical traineeship experience at *La Maison Bleue*. Data collected from key informants allowed (a) a better understanding of the management and logistical organization of traineeships at *La Maison Bleue* and (b) comparison and validation, through triangulation methods, of factual information and perceptions reported and described by physicians.

First author (JM) conducted all the semi-structured individual interviews between September 18, 2017 and April 26, 2018. Physicians participated in a single interview lasting about 60 min. Five of the twelve interviews were conducted in person. The participant was free to determine the location he or she deemed appropriate for the interview. In only one of these cases did the interview take place at the participant’s workplace. The other four face-to-face interviews were conducted in a coffee shop chosen by the participant. In cases where a face-to-face meeting could not be arranged, the interviews were alternately conducted through an online mode (*n = 5*) or by telephone (*n = 2*). All interviews were recorded with the participants’ consent. JM was also responsible for the transcription of all interviews. The recordings and transcripts were made available to SD and EM (research directors) for reference.

Participation was voluntary. All participants were explicitly informed that they remained free at all times to withdraw from the project or refuse to answer certain questions asked during the interview, without prejudice. No participants actually withdrew from the study or refused to answer questions. Particular attention was paid to maintaining the confidentiality of the data collected. Ethical approval for this study was issued by the West-Central Montreal CIUSSS Research Ethics Committee in May 2017 (17–043 31-05-2017) and by Université Laval Research Ethics Committee in July 2017 (2017–193 25-07-2017).

### Data analysis

Qualitative content analysis was conducted following L’Écuyer’s guidelines [48]. As proposed by the author [48], given the small sample size (*n* = 12), the data were not quantified to avoid low, unstable and insignificant frequencies. The analysis began with a preliminary reading of all the data in order to dwell in and familiarize oneself with the analytical material, identify a number of ideas evoked by the data corpus and then identify the first information units to be used for the subsequent classification of the material. In a first step, an open coding strategy aimed at “breaking down the material into smaller segments each holding a complete meaning in and of itself” was used (p. 59). The next step was to formulate codes to summarize the central idea of these units of meaning. The approach to coding was mostly inductive to ensure the primacy of participants’ voices, while ensuring consistency with the research objectives. Otherwise, as “concepts and findings identified in earlier literature may increase and help guide inductive qualitative content analyses in useful ways” (p. 106), we kept in mind the key elements of our literature review, the central elements of our interview guide and Mezirow’s theory of transformational learning [41–43] when creating the coding grid [49]. Finally, the iterative analytic process involved category building reflecting statements’ meaning similarities, code additions and eliminations, and revision of the coding hierarchy. JM was the main responsible for the analytical process. However, the codification of a first transcript was done jointly by JM and EM in order to agree on a strategy and preliminary grid. Subsequently, JM reported regularly to EM and SD on the status of her analytical work and incorporated their comments and suggestions to strengthen the description of emerging analytical categories and to gradually improve the coding strategy and coding grid. NVivo software was used to carry out the coding.

## Results

### Participants’ socio-demographic and professional profile

Our 12 participants all have 4 years or less of practice experience since graduation. In addition, most have 2 years or less of experience (*n* = 9). These are young physicians, most of them between the ages of 25 and 29 (*n* = 8). Also, the vast majority of our participants are women (*n* = 10). Most of our participants currently practice exclusively in urban areas (*n* = 8), the others have a rural practice (*n* = 1) or a mixed urban-rural practice (*n* = 3). Finally, the majority of participants (*n* = 10) practice in FMGs (Family Medicine Groups) or FMG-Us (Academic Family Medicine Groups).^3^ The remaining work either in a front-line setting elsewhere in Canada, or in a hospital setting.

Most participants mentioned that they had an interest in community and global health, and/or in populations experiencing social vulnerability, prior to their traineeship at *La Maison Bleue* (*n* = 10). For many, this interest had translated into concrete experiences during their medical training. For example, some participants had completed a university microprogram in international health, while others had participated in international traineeships to improve their understanding of issues faced by vulnerable populations abroad (e. g. in Haiti, Africa, Indonesia). Others had had traineeship experiences (e.g. in social paediatrics, with indigenous populations, in disadvantaged neighbourhoods, in rural areas) or volunteer experiences (e.g. Doctors Without Borders) among vulnerable populations.

Apart from the participating physicians, the three key informants interviewed to gain insight on the nature and organisation of the traineeship were from *La Maison Bleue* (*n* = 1), from a FMG-U involved with *La Maison Bleue* in organizing traineeships (*n* = 1) and from the academic community (*n* = 1).

### Characteristics of the traineeship at La Maison Bleue

For most participants, choosing *La Maison Bleue* as a traineeship setting was a personal initiative (*n* = 10). Otherwise, participants who indicated that this traineeship setting was imposed on them by their program administrators (*n* = 2) reported that this decision was made in accordance with their known interests in working with vulnerable populations or in community practice.

Participants who completed their traineeship during preclinical training or clerkship reported more of an observational experience. Otherwise, residency and fellowship level traineeships reported a combination of observation and active clinical participation, under the guidance of a supervising physician.

Table 1 presents the main characteristics of the traineeship experience as reported by participants.

**Table 1:** Main characteristics of the traineeship experience

		N
Context and motivations for the choice of *La Maison Bleue* as an traineeship setting	Personal choice	10
	Allocation by a third party	2
	Total	*12*
Timing of the traineeship	Preclinical only	1
	Residency only	2
	Fellowship only^a^	6
	Preclinical AND residency (2 interships)	1
	Clerckship AND residency (2 interships)	2
	Total	*12*
Traineeship duration^b^	0–7 days	5
	8–14 days	3
	15–21 days More than 21 days	3 1
	Total	*12*

^a^These students (participants in an advanced skills programs in maternal and child health care) are characterized by the fact that they had completed their first two years of residency in family medicine at the time of the traineeship at *La Maison Bleue* and therefore had their medical license in hand

^b^We note that the duration of the traineeship is a factor that is highly variable depending, for example, on the traineeship program from which the learner came, the time of the year at which the traineeship was completed, or the philosophy of the supervising physician. In fact, none of our 12 participants has the same traineeship arrangements as another. This finding is supported by what key informants said about the organization and general terms and conditions of the traineeships being, in several cases, decided on a case-by-case basis and not documented. Thus, we consider that the number of days of traineeship indicated in Table 1 does not even adequately reflect this variability since, for example, a 5-day traineeship may have been full-time for one or 1 day per week for 5 weeks for the other

### The learnings gained

All participants considered that the experience was, on various levels, conducive to new learnings. As they identified certain limitations (e.g., lower caseloads, limited resources, work space constraints) to developing specialized clinical and technical skills, a vast majority of participants stated the main learnings occurred on a human, social and relational level.

Four main categories of learning were identified from the analysis: [1] greater awareness of beliefs, assumptions and biases [2]; the development of critical perspectives regarding the health care and social services system [3]; a renewed vision of medical practice; and [4] strengthened professional identity and future practice orientation.

#### Greater awareness of beliefs, assumptions and biases

According to participants, the traineeship experience in a community-based setting serving people experiencing circumstances of social vulnerability led to a new sensitivity to the diversity and complexity of life trajectories. It also led to a new understanding of how each unique patient’s trajectory may constrain or foster individual choices and behaviors, and ultimately have an impact on health. Furthermore, the traineeship constituted an opportunity to become aware of certain variances in norms, values, knowledge, and practices regarding perinatal and family health, as illustrated here:

*I remember the first little Pakistani babies I saw. They were all made up.*^4^*There you say to yourself: "Oh boy! I'm not sure it fits my frame of reference!" I thought I was an open-minded person… but you get there [at La Maison Bleue] and then you understand that there are many other ways to see things. (Participant A)*

Participants also highlight that the experience led to a certain reflexive self-awareness through the observation that their status (e.g. physicians, Canadians, from wealthy families) comes with its share of power and privilege, as stated in these words by a participant:

*As doctors, we are all very fortunate in life, we have to know how to get out of that context and be able to see that there is something else and how difficult it can be, how it can be challenging to be in other situations that aren't like ours. (Participant B)*

#### The development of critical perspectives regarding the health care system

Some participants also mentioned that they were able, based on their experience at *La Maison Bleue*, to deepen their analysis of the health system and to consequently gain a better understanding of the challenges it faces and its weaknesses in meeting the needs of the most marginalized and vulnerable. Participants who addressed this point supported arguments fostering the system’s openness to initiatives better adapted to the patients’ needs, at the local and community level.

#### A renewed vision of medical practice

According to many participants, the traineeship fostered the questioning of classical intervention models, by introducing less stigmatizing approaches fundamentally based on listening, patience, openness, empathy, and deep respect for the human being as a whole, as illustrated here:

*I really became aware of the power of that tool, just to sit down, shut up, and listen. (Participant A)*

The experience also raised participants’ awareness of the importance of psychosocial support (what they refer to as advocacy) to help patients access social and community-based resources throughout their health trajectories and, consequently, to improve their well-being through upstream action on social determinants of health. Comparable importance was given to the adoption of an empowering practice supporting patients in the development of renewed self-esteem, awareness of their abilities and possibilities for taking action and regaining control over their own lives.

Also, several participants mentioned that during their traineeship at *La Maison Bleue*, they became aware of the added value of interprofessional practice. According to the many physicians met, participation within *La Maison Bleue*’s interdisciplinary team and contact with role models having expertise and know-how for the care of people experiencing social vulnerability, allowed them to develop in-depth knowledge and understanding of the roles and mandates of other professionals and their contribution to the global care process. Besides fostering interprofessional confidence and respect, participants report this learning led to a recognition of the limits of one’s skills as a physician and a reflection on one’s role on the team:

*As a physician you have to understand that it's not necessarily your role to do everything. There are other people better trained for that. (Participant C)*

Similarly, participants mentioned they realised, through their traineeship experience, how interdisciplinary practice optimizes problem-solving and allows the burden of managing complex cases to be shared among team members, thus reducing a certain feeling of helplessness, as expressed by a participant in the following words:

*In medicine, you witness situations of extreme vulnerability and then you find yourself without satisfying solutions. It [the traineeship experience at* La Maison Bleue*] made me realize that I'm not alone, and there's a way to take care of these people. (Participant A)*

Physicians also outlined that the traineeship was for them an opportunity to develop better knowledge and understanding of community-based services, to better appreciate the relevance of developing and maintaining a strong link with community-based organizations, and of using these resources wisely and frequently in the delivery of care to people experiencing circumstances of social vulnerability.

#### Strengthened professional identity and future practice orientation

Most participants mentioned that the experience did crystallize in their minds an ideal of front-line care they wished to replicate in their future practice. Accordingly, the experience was outlined by some participants as contributive to professional identity development. It thus confirmed community-based professional practice as some participants’ professional niche. Participant A for whom the traineeship experience was an opportunity to discover an approach to care in perfect coherence with her personal profile and professional expectations explained it as follows:

*I felt carried away. I felt that I had the right to be myself. It's [the traineeship experience at* La Maison Bleue*] really come to meet a deep need for correspondence between who I am as a person and how I can serve the public. (Participant A)*

Likewise, according to some participants, the experience led to the definition of a set of essential criteria for the selection of their future practice environment so it fits their values, expectations and professional objectives. Participants mentioned that, following their traineeship at *La Maison Bleue*, they stated their willingness to practice in small care settings, promoting interprofessional practices, and serving marginalized or disadvantaged populations. For some, the formulation of these criteria was also linked to their willingness to influence organisational and social change. Comments of a resident interviewed illustrate how the traineeship experience has the potential to impact the orientation process:

*Clinics I’m interested in are places that are a little smaller, where my word is going to be heard, where I will have more control over the decisions made. (Participant D)*

Moreover, for some, the traineeship initiated a willingness to become, as a physician, an actor of social change through the promotion of new ways of thinking, being and doing, and advocacy for more human values in medical practice. For instance, some participants mentioned that the traineeship experience contributed to an awareness of the determinants of inequities in access to health care and, consequently, of the importance of getting involved, as a physician wanting to defend the values of access and equity, as stated in these words by a participant:

*There have been patients in particular who have shown me that they do not have the same resources as I do and that there are great inequalities in the way we can manage our own health. (Participant E)*

### Characteristics of the traineeship experience that influenced the learnings

Globally, most physicians interviewed mentioned the singularity of the traineeship experience (in comparison with past classical traineeship experiences) and the special type of organization *La Maison Bleue* constitutes. Accordingly, several participants qualified the experience as potentially frustrating and destabilizing, but also a way to better understand the relevance of the alternative approach to care. A participant’s comments illustrate this idea of relevance:

*It's organized chaos, but it's part of the game. It's part of what it's like to work in a community-based clinic where there are people with needs that don't fit into the little time boxes you get when you're in a regular clinic. (Participant F)*

That being said, analysis allowed us to outline three main factors which are associated with previously outlined learnings: [1] upstream factors linked to the learner’s individual profile and trajectory, [2] factors linked to *La Maison Bleue* setting and the traineeship modalities, and [3] factors linked to the clientele’s profile.

#### Factors linked to the upstream learner’s individual profile

Participants outlined the learner’s individual profile and trajectory (e.g. past experiences, personality, values, interests, needs, objectives) as a factor influencing the levels of commitment and curiosity necessary to actually benefit from the new knowledge and skills accessible through the traineeship experience. For most participants, their individual profile seemed to have fostered a memorable experience. However, for one of them who completed his traineeship as a preclinical student, the lack of previous clinical experience contributed to limiting his propensity to attend to and learn from the specific context and issues related to social vulnerability:

*It was a time when I was very receptive, but what I was learning was very very basic things about the medical interview. (Participant G)*

#### Factors linked to *La Maison Bleue* and the traineeship modalities

Most participants suggest that interdisciplinarity, as experienced at *La Maison Bleue*, where teamwork is perceived as implemented in a unique way, is a fundamental source of learning throughout the experience. Only two participants provided nuances about the uniqueness of this interdisciplinarity*,* as they considered interdisciplinary practice as more and more frequent and valued in a growing number of health care settings.

Thus, most participants outlined the value of observing other professionals’ approach to care and interactions with other members of the interprofessional team throughout their learning processes. Concretely, several physicians attribute this great value to the openness and active commitment of *all* professionals to learners’ training and to *La Maison Bleue’s* approach. As such, the training environment, according to participants, provides constant exposure to various perspectives, to positive role models, and to the values of active welcoming and inclusion of learners into a context of non-hierarchical interdisciplinary and shared responsibility for care, as illustrated here:

*In the hospital, the hierarchy is very, very strong. It's hard. You feel judged all the time and it makes your learning heavy. At* La Maison Bleue*, it's not like that at all, you don't have the weight of the hierarchy, you don't have the weight of judgment either. As a learner, you just want to develop and flourish. (Participant A)*

Moreover, some characteristics of *La Maison Bleue*’s particular social and organisational model were also mentioned as contributive to learnings. Notably, participants highlighted the informality, ease and frequency of interprofessional exchanges. The latter were lauded for the flexibility and adaptability allowed in terms of time, space and approach, and the physical organization of the premises were praised for promoting physical proximity and a friendly atmosphere. A participant, who now practices in a primary care clinic integrated within a major hospital setting, speaks of physical organization and its repercussions on medical practice:

*The contacts were very easy. We're in a house, we're each in our own room. You can go knock on the neighbour's door and ask your question. At my clinic, I start my day, I arrive at 8:00, I don't talk to anyone, I see my patients. (Participant B)*

Also, several participants identified interdisciplinary meetings held daily as an important learning opportunity. Only one participant indicated not being very involved in this process because it mostly dealt with unfamiliar cases. Otherwise, most consider these meetings as a pillar of interdisciplinary practice at *La Maison Bleue* and perceive them as a space for sharing experiences and ideas, and for defining common orientations.

#### Factors linked to the clientele profile

Finally, making direct interpersonal contact with *La Maison Bleue*’s clientele—generally characterized by the complexity of its migration path, by a situation of social and material disadvantage and by the experience of several barriers to health care access—was also considered an important influence. According to participants, direct contact with the patients ‘put a face’ on the idea of social vulnerability. Furthermore, opportunity to observe the impact of *La Maison Bleue*’s team approaches and actions on these people’s life trajectories and particular needs was mentioned as contributive to learnings about the relevance of the approach. A participant gave an example of such an impact, on a social level:

*What I found most different was how well people felt when they came to* La Maison Bleue*. People would come in just to have a glass of water or just to sit in the kitchen. It offered this door that is always open, which is not available in other clinics. (Participant C)*

## Discussion

The findings drawn from this study on the learning gained from a traineeship experience at *La Maison Bleue* converge with the literature in that they identify clinical training as a central place for the acquisition of knowledge, skills, attitudes and behaviours [17, 50, 51]. However, our results suggest this particular traineeship experience fosters a shift in perspectives away from the medical model to which learners are exposed through more classical traineeship settings–which often rely more on a curative approach and a biomedical discourse, with little regard for the social determinants of health. In this sense, the community-based experience represents an avenue of particular interest for fostering transformative learning as proposed by Mezirow [41–43]. Indeed, the learning model proposed by *La Maison Bleue* presents with several of the central characteristics identified by Hirsh and colleagues [52] as important for structuring training models consistent with a transformational approach: training based on interpersonal relationships, on the conception and actualization of authentic roles, and on significant correspondence with ideals of medicine such as service, advocacy and a patient-centred approach. Moreover, the fact that some participants mention the potentially frustrating and destabilizing nature of the experience appears indicative of a certain gap between what is experienced and the usual mental frameworks valued through classical medical training. Following Mezirow [41–43], such a gap has the potential to initiate the transformational learning process. Our results are in line with those of van den Heuvel and colleagues [53], who suggest that such a gap is often catalyzed by proximity to a clientele in a situation of extreme social vulnerability as well as by interactions with a competent interdisciplinary team in which trainees often found role models deemed inspiring in terms of values, attitudes, behaviours and practices. As part of their experience at *La Maison Bleue*, learners would thus have been prompted to critically reassess their presuppositions regarding the objectives to be pursued in the practice of primary care medicine, to question the capacity of the health and social services system to respond to patients’ needs and expectations, and to explore new alternatives. Drawing on Mezirow’s theoretical perspective [41–43], these alternatives are what we call transformational learning.

The following section discusses in more details the transformational learning opportunities offered by settings operating under an alternative paradigm and their potential role for medical practice transformation toward access-to-care and health equity.

### Learning through reflexive introspection

Our results suggest that the traineeship experience within a community-based setting like *La Maison Bleue* can lead certain learners to become aware of their prejudices and assumptions about physician’s professional practice and role on a pragmatic level, what authors refer to as a formative reflexivity aimed at improving professional practice [54]. However, as proposed by Tremblay and colleagues [54], our results also suggest the development of a more critical reflective process, which sits beyond the formative exercise and on which important learnings are based. Indeed, as documented elsewhere [34, 53, 55], our analysis revealed learners involved in such an experience can develop a reflexive awareness of the social distance that exists between them and the patients served at *La Maison Bleue* that is contributive to social competence (i.e. knowledge, skills and attitudes that support effective physician-patient interaction despite the intervening social distance [56]). Indeed, consistent with the work of Loignon and colleagues [55], our result suggest the traineeship experience would, in a perspective of deconstructing prejudices, be an opportunity for learners to become aware of and reflect on their prejudices and misperceptions about what social vulnerability is and what it implies in everyday life for those experiencing it. According to Loignon and colleagues [55], such critical reflection has the potential to result in the implementation of less stigmatizing practices since it is enriched by a new social competence. Following Tremblay and colleagues [57], this learning is predicated on a relational reflexivity, focused on the development of an empathetic, healthy and effective therapeutic relationship, that allows the professional to act with compassion and lucidity towards his patients. As observed in our study, authors [57] suggest such reflexivity results from the examination of emotions, values, beliefs, bias and prejudice related to patients and clinical situations, the explicitation of non-rational elements that are underlying judgments and practices, and the recognition of the influence of these elements on building better therapeutic relationships.

Further, while they indicate that the traineeship has fostered the questioning of dominant healthcare practices and has contributed to the desire, for some physicians, to position themselves as agents of organizational and social change and advocate against health inequities, our results suggest that the reflexive learning could go beyond the patient-doctor dyad. Indeed, it could contribute to the questioning of the premises of medical practice and the social and structural issues associated with it. In that sense, as outlined by other authors [34, 58], our results suggest the traineeship experience might be an opportunity to gain a renewed understanding and awareness regarding the social determinants of health and to develop a will to take action upon them. Further, the experience might foster a ‘structural competency’ that “emphasizes diagnostic recognition of the economic and political conditions that produce health inequalities in the first place” and calls on healthcare providers “not only to recognize how institutions, markets, or healthcare delivery systems shape symptom presentations but also to mobilize for correction of health and wealth inequalities in society” (p.460) [59].

### Learning through social interactions

Beyond introspective reflexivity, the fact that thinking, acting and feeling as a physician is gradually realized for medical students within a dynamic system of shared and internalized values, symbols, norms, narratives, and concepts, has been documented elsewhere [60–62]. Our results report this social dimension of learning as they suggest a relational commitment with other professionals, bearers of different ways of doing, being and thinking. Indeed, the results propose that the integration of learners into interprofessional practice, enriched by complementary points of view in a distinctive non-hierarchical framework for sharing ideas and knowledge, represent an important factor promoting new questionings and understandings regarding medical practice. Moreover, collective modes of supervision that prevail in such a context, while opposing the traditional conceptualization of medical traineeship supervision based on the supervisor’s formal authority over learners [63], would (a) allow the trainee to access complementary knowledge and expertise, particularly in the psychosocial sphere and (b) relieve the learner of the heavy responsibilities, associated with the medical power as well as the fear of judgment of others that seems to prevail particularly in the hospital setting [63]. Indeed, our results converge with the findings of other studies suggesting that in an interprofessional setting, the trainee would become more willing to learn from observation and interaction with role models and more engaged in seeking new information, advice and feedback [63].

Finally, the results show that some trainees have, through their experience, confirmed their interest in community-based professional practice, in small care settings, promoting interprofessional practice, with marginalized or disadvantaged populations. Other authors have also established a link between traineeship experience in non-traditional settings and professional intentions and orientations [31, 32]. We see the expression of these intentions and orientations as contributing to the development of professional identity in that they constitute, in our view, the participants’ articulation of the impact of the traineeship experience on their future as physicians.

### Limitations and agenda for future research

Our study is not without limitations. First, we have taken into account the voice of 12 people out of a few hundred trainees. It is therefore reasonable to think that those who wanted to participate were those who are closer to the organisation, share its values and particularly loved their traineeship experience. This premise is further supported by the fact that the vast majority of our participants (*n* = 10) made a deliberate choice to do a traineeship at *La Maison Bleue*, whereas for others (*n* = 2) assignment to *La Maison Bleue* for their traineeship was in accordance with their interests for practice with vulnerable populations and/or community-based practice. This suggests that most participants were potentially predisposed to achieve the learnings identified. Some people may have had a less positive traineeship experience at *La Maison Bleue* or have been exposed to this setting with little or no prior interest for practice with vulnerable populations and/or community-based practice. We unfortunately did not get their input into this study. This deprives us of information that could have counterbalanced the results obtained, which generally report relevant learning and a willingness to apply them to front-line practices.

Also, it is interesting to note that reflexive thinking during the traineeship at *La Maison Bleue* is activated autonomously by the trainees. Indeed, to our knowledge, contrary to the experience reported by Loignon and collaborators [55] where structured reflexive activities were proposed to learners during their traineeship, no such activities were proposed during the traineeship at *La Maison Bleue*. This observation does not exclude that a structuring of the physical environment or a particular organization of the workflow at *La Maison Bleue* may to some extent have stimulated the reflexivity of certain trainees during their learning experience. We can therefore assume, in light of the results, that many of the participants we met had individual characteristics or skills that predisposed them to take advantage of aspects of the context so as to question themselves about their prejudices and bias, their practice and the associated social issues. One might also wonder about the potential catalyzing effect that a structured reflexive activity could have had during the traineeship at *La Maison Bleue*. We consider this could be further investigated.

Moreover, the relatively homogeneous small sample has limited our ability to assess the influences of different individual characteristics (e.g. gender, age, time since graduation) on the variability of participants’ perceptions and experiences. Also, the fact that the traineeship lasted, in many cases, only a few days led us to question whether a traineeship of such a short duration has the potential to generate fundamental learning. For instance, the literature suggests a positive link between the duration of exposure to a practice environment located in an underprivileged area and the development of particular knowledge, attitudes and skills regarding the burdens and challenges faced by communities [31, 33]. That being said, such a relationship could not be clearly established in the context of this study, since some participants who had completed a short traineeship also identified significant learnings. Again, we postulate that these learnings may stem from their prior commitment and openness, a predisposition that itself stems from their origins, values and past experiences. Indeed, our results highlighted that many of our participants had an interest and experience in community or international health prior to their traineeship at *La Maison Bleue*. Results also suggest that the timing of the traineeship within the medical training course may influence the learners’ predisposition to the learnings identified. This suggests the link between the duration of exposure and the development of fundamental learnings may present a certain level of complexity since it may be influenced by a multitude of other factors. It may therefore be further investigated.

Other avenues for future research were also identified. First, we consider it would be relevant to reproduce this study in multiple alternative settings in a comparative perspective in order to further inform our understanding of how different local characteristics may influence the learnings generated. Also, Tamuz and colleagues [63], to whom we referred above, explored the effects of medical hierarchy on resident engagement in the learning process in the specific context of intensive care units where traditional supervision and interprofessional supervision overlap. To our knowledge, this issue has not been addressed in a community context and could therefore be the subject of future research. Finally, in the current Quebec context of constraining and highly standardized conditions of medical practice, we consider of particular interest (a) to document whether physicians who completed a traineeship in an ‘alternative’ setting and who therefore wish to practice medicine differently, are able, beyond intentions, to actually reinvest the learning gained into their front-line practices and (b) to identify the strategies adopted, as well as the facilitating factors and barriers encountered in their attempts to do so.

## Conclusion

This study sheds light on how, through their particular experience within a clinical setting intended for people experiencing circumstances of social vulnerability and operating under an alternative paradigm of care, medical learners came to reflect on their own beliefs, assumptions, and biases; to critically examine the health care system and its capacity to meet diverse populations’ needs; to renew their vision of medical practice; and to develop their professional identity. It contributes important knowledge to the medical education literature, helping to fill a gap and shedding new light on how community-based traineeship experiences can potentially lead to the development of fundamental learnings that are conducive to acceptable and equitable care for people experiencing social vulnerability. This article therefore deals with issues that are at the heart of public health concerns about health equity, notably those concerning the development of measures to promote equitable access-to-care for people experiencing circumstances of social vulnerability. Furthermore, through a qualitative analysis of the learning physicians associate with their community-based traineeship experience, this study presents novel insights into traineeship characteristics that may foster those learnings. Its originality also lies in its understudied research setting. We consider that revealing its distinct characteristics may foster innovative future research and provide avenues to be explored for the renewal of practices, to ultimately allow physicians to better address the contemporary challenges of medical practice [64], and support academic institutions’ commitment to social responsibility and sustainable population health [65]. Though such contextualized study results may arguably be considered not generalizable, our view is that the knowledge generated may be applicable and pertinent to foster a reflection regarding traineeship experiences in similar contexts.

## Data Availability

The datasets used and/or analysed during the current study are available from the corresponding author on reasonable request.

## Abbreviations

CIUSSS: Centre intégré universitaire de santé et de services sociaux
FMG: Family Medicine Group
FMG-U: Academic Family Medicine Group
R3: Third year of a medical residency program

## Footnotes

1 Quebec is an eastern Canadian province. In Canada, roles and responsibilities for health care services are shared between the federal government and provincial and territorial governments. Provincial and territorial governments are responsible for the management and organization of health care services. In addition, they provide these services to their residents. In Quebec, the health system is public, with the State acting as the main insurer and administrator. Two universal regimes allow the entire population to obtain hospital and medical services at the expense of the State: the Hospitalization insurance plan (1961) and the Health insurance scheme (1970). In addition, some services are offered free of charge to specific groups, based on specific criteria. In addition, in 1997, the General drug insurance plan supplemented the public coverage of Quebec’s population. It is a universal blended plan, based on a partnership between the government and private insurers [6]

2 *La Maison Bleue* is operating under the status of a charity and non-profit organization, linked to the Quebec formal health and social services network by agreements with the CIUSSS of the territory. Three sites are in operation at the time of writing this article: *La Maison Bleue de Côte-des-Neiges* and *La Maison Bleue de Parc-Extension* which are both attached to the *Centre-Ouest-de-l’Île-de-Montréal* CIUSSS and then to *La Maison Bleue de Saint-Michel*, attached to the *Est-de-l’Île-de-Montréal* CIUSSS. Although they are not themselves considered FMG-U (FMG responsible for the provision of clinical training to medical learners) sites given the fact that their mission is limited to perinatal care and do not cover the whole spectrum of family care, the three *Maisons Bleues* are all part of a FMG-U (Maisonneuve-Rosemont for the *Maison Bleue de Saint-Michel*, Village Santé for the *Maisons Bleues* de Côte-des-Neiges and Parc-Extension). Thus, the doctors who work in the *Maison Bleues* are also clinical professors of the FMG-Us in the two CIUSSS.

3 Main front-line care organizational model within the Quebec health and social services network since 2000.

4 Some practices, such as those noted here, refer to symbolism, rites and customs specific to certain cultural groups. In some countries, for example, the baby’s eyebrows and eyelids are kohled to strengthen the power of the eye. Also, sometimes, dots are drawn on babies’ faces.
